# Epigenetic Changes Governing *Scn5a* Expression in Denervated Skeletal Muscle

**DOI:** 10.3390/ijms22052755

**Published:** 2021-03-09

**Authors:** David Carreras, Rebecca Martinez-Moreno, Mel·lina Pinsach-Abuin, Manel M. Santafe, Pol Gomà, Ramon Brugada, Fabiana S. Scornik, Guillermo J. Pérez, Sara Pagans

**Affiliations:** 1 Cardiovascular Genetics Center, Biomedical Research Institute of Girona, 17190 Salt, Spain; dcarreras@gencardio.com (D.C.); rmartinez@gencardio.com (R.M.-M.); mpinsach@gencardio.com (M.P.-A.); polgomamas@gmail.com (P.G.); rbrugada@ldibgi.com (R.B.); 2 Department of Medical Sciences, Universitat de Girona, 17003 Girona, Spain; 3 Unit of Histology and Neurobiology, Department of Basic Medical Sciences, Faculty of Medicine and Health Sciences, Rovira i Virgili University, 43003 Reus, Spain; manuel.santafe@urv.cat; 4 Centro de Investigación Biomédica en Red de Enfermedades Cardiovasculares (CIBERCV), 21005 Madrid, Spain; 5 Hospital Josep Trueta, 17007 Girona, Spain

**Keywords:** cardiac arrhythmias, *Scn5a*, transcriptional regulation, epigenetic mechanisms, skeletal muscle denervation, histone modifications, H3K27 acetylation

## Abstract

The *SCN5A* gene encodes the α-subunit of the voltage-gated cardiac sodium channel (Na_V_1.5), a key player in cardiac action potential depolarization. Genetic variants in protein-coding regions of the human *SCN5A* have been largely associated with inherited cardiac arrhythmias. Increasing evidence also suggests that aberrant expression of the *SCN5A* gene could increase susceptibility to arrhythmogenic diseases, but the mechanisms governing *SCN5A* expression are not yet well understood. To gain insights into the molecular basis of *SCN5A* gene regulation, we used rat gastrocnemius muscle four days following denervation, a process well known to stimulate *Scn5a* expression. Our results show that denervation of rat skeletal muscle induces the expression of the adult cardiac *Scn5a* isoform. RNA-seq experiments reveal that denervation leads to significant changes in the transcriptome, with *Scn5a* amongst the fifty top upregulated genes. Consistent with this increase in expression, ChIP-qPCR assays show enrichment of H3K27ac and H3K4me3 and binding of the transcription factor Gata4 near the *Scn5a* promoter region. Also, Gata4 mRNA levels are significantly induced upon denervation. Genome-wide analysis of H3K27ac by ChIP-seq suggest that a super enhancer recently described to regulate *Scn5a* in cardiac tissue is activated in response to denervation. Altogether, our experiments reveal that similar mechanisms regulate the expression of *Scn5a* in denervated muscle and cardiac tissue, suggesting a conserved pathway for *SCN5A* expression among striated muscles.

## 1. Introduction

Voltage-gated sodium channels (VGSC) are key players in cell excitability. The VGSC gene family is formed by nine homologous members, *SCN1A*–*SCN11A*. These genes encode nine channel α-subunit isoforms named Na_V_1.1 to Na_V_1.9. These isoforms are differentially expressed across species and also in tissues of the same species [1]. VGSC differ in their electrophysiological properties, and sensitivity to neurotoxins such as tetrodotoxin (TTX) and saxitoxin [2].

The *SCN5A* (formerly SkM2) gene encodes the α-subunit of the voltage-gated cardiac sodium channel (Na_V_1.5), which plays a key role during the depolarization phase of the cardiac action potential. The Na_V_1.5 protein has 2016 amino acids and a calculated molecular weight of 227 kDa. As all VGSC, Na_V_1.5 consists of four homologous domains, known as DI to DIV, joined by so-called intracellular linkers [3]. This pore forming α-subunit is typically associated with one or more auxiliary β-subunits [4,5]. *SCN5A* is mainly expressed in the auricular and ventricular muscle, although it has also been reported to be expressed in other tissues. These include neonatal dorsal root ganglion neurons [6], human intestinal smooth muscle and Cajal cells, [7,8] neurons from distinct regions of the brain [9,10], as well as intracardiac neurons [11].

Skeletal muscle constitutes a special case for *SCN5A* expression. In this tissue, *SCN5A* mRNA is expressed early in the postnatal development, but its expression declines with age as the expression of *SCN4A*, encoding the Na_V_1.4 channel, increases. However, re-expression of Na_V_1.5 can be readily induced by skeletal muscle denervation [12]. In fact, although Na_V_1.5 is the predominant sodium channel in the heart, the elucidation of its molecular identity has been tightly associated with research in denervated skeletal muscle. Pioneering observations from early 70′s identified the development of TTX-resistant action potentials in mammalian skeletal muscle upon denervation [13,14,15]. This component was hypothesized to be the product of a new population of sodium channels induced by denervation [16]. A decade later, this hypothesis was confirmed by demonstrating that the previously cloned *SkM2* gene produced TTX-resistant Na^+^ currents when injected into *Xenopus* oocytes. *SkM2* not only produced TTX-resistant Na+ currents but its DNA sequence was identical to the sodium channel sequence found in cardiac muscle [12,17,18]. Several studies have examined the expression of the TTX-resistant *Scn5a* sodium channel in denervated skeletal muscle. Based on the difference in TTX sensitivity between Na_V_1.4 and Na_V_1.5, Sellin and Thesleff [19] showed that *Scn5a* expression reaches a peak 3–5 days following denervation. In a separate study, Yang et al. [20] showed that following denervation, *rSkM2* (*Scn5a*) mRNA levels rapidly increased in the period from 24 to 72 h, and then started to decline. Soon after, this group cloned the promoter region and identified key regulatory elements (RE) for the transcriptional regulation of *SCN5A* [21]. Whilst Na_V_1.5 expression is absent in innervated muscles [16], its known up-regulation upon denervation has been used in electrophysiological and immunohistochemical assays to quantify the loss of muscle innervation in aging [22,23]. More recently, Sekiguchi et al. [24] demonstrated that Na_V_1.5 expression is associated to the typical pathophysiological appearance of fibrillation potentials seen in muscle denervation.

The clinical relevance of Na_V_1.5 can hardly be emphasized as defects in the *SCN5A* gene are closely associated to lethal cardiac arrhythmias. *SCN5A* was the first gene associated with Brugada syndrome (BrS) and still remains as the major gene linked to BrS pathogenesis [25]. A series of single nucleotide variants as well as small insertions and deletions in exonic regions of the *SCN5A* gene have been largely associated with Na_V_1.5 loss-of-function and have been directly linked to 11–24% of BrS cases [26]. Exonic variants in the *SCN5A* gene have also been linked to other life-threatening cardiac arrhythmias such as long QT Syndrome type 3, atrial fibrillation, or sudden infant death syndrome [27].

During the last years, increasing evidence has suggested that alterations in *SCN5A* gene expression may also increase susceptibility to arrhythmogenic diseases. For instance, mice expressing a single copy of the *Scn5a* gene show cardiac defects that resemble those observed in humans with BrS [28]. Work from Bezzina and colleagues also demonstrated that certain haplotypes in the *SCN5A* promoter associated with changes in *SCN5A* transcriptional activity are also linked to variations in the QRS electrocardiogram interval [29]. Another line of evidence comes from the work from van den Boogaard and colleagues, who showed that a genetic variant in a distal *cis*-regulatory region affecting the binding of transcription factors (TF) TBX3/TBX5 and *SCN5A* gene expression is associated with cardiac conduction disease [30,31]. Collectively, these findings suggest that dysregulation of *SCN5A* gene expression is linked to cardiac disease, and that understanding the regulatory mechanisms of *SCN5A* gene expression is critical to uncover novel etiologies of Na_V_1.5-related arrhythmias. In this sense, during the last years, there have been several reports focusing on the study of the mechanisms that regulate *SCN5A* expression. For instance, our group uncovered that the TF GATA4 synergizes with GATA5 to activate transcription of the *SCN5A* gene [32]. GATA4 has also been shown to act cooperatively with IRX5 in the regulation of *SCN5A* gene expression [33]. However, the role of epigenetic modifications on *SCN5A* gene regulation is not completely understood. The well documented functional expression of Na_V_1.5 channels in denervated muscle prompted us to use this experimental paradigm to study the epigenetic changes underlying this phenomenon.

Denervation of skeletal muscle offers the advantage of comparing gene expression and epigenetic modifications in denervated versus non-denervated, contralateral muscles from the same animal. Therefore, it appears as a suitable model to study epigenetic changes associated with *SCN5A* expression. Here, we used the denervated gastrocnemius from rat to study the differential expression of *Scn5a* and examine the transcriptomic and epigenetic changes associated with denervation. Our results provide evidence for a unified view of the general epigenetic mechanisms that govern the expression of *SCN5A* in both heart and denervated skeletal muscle.

## 2. Results

### 2.1. Denervated Gastrocnemius Muscle Expresses the Scn5a Adult Isoform

Abnormal re-expression of *Scn5a* upon denervation of skeletal muscle has long been described but, up to date, the *Scn5a* isoform that is expressed in these conditions was still unknown. Different splicing isoforms have been described for *SCN5A*. For instance, during cardiac development, there is a splicing switch, in which fetal exon 6A is replaced by the adult exon 6B after birth. Exons 6A and 6B are mutually exclusive and encode a segment of the voltage sensor (segments 3 and 4 from DI).

**Figure 1:**
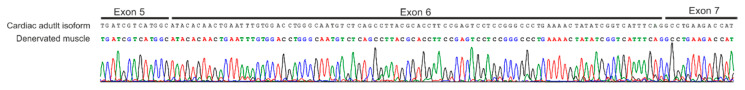
Comparison of exon 6 sequence between reference cardiac adult isoform (top sequence) and the sequence obtained from the denervated skeletal muscle (bottom sequence). The electropherogram corresponds to the product obtained for the denervated muscle.

To determine the *Scn5a* isoform expressed in response to denervation, we purified total RNA from rat gastrocnemius muscle, obtained four days after denervation. After reverse transcription and amplification with specific primers (Table S1), we determined the complete *Scn5a* cDNA sequence by Sanger sequencing. Comparison of the sequence obtained from the denervated muscle with the *Scn5a* adult cardiac sequence, taken as reference, showed 100% identity. More specifically, we observed that the *Scn5a* sequence from the denervated muscle is identical to the adult cardiac type exon 6B, in which the adult and neonatal forms differ (Figure 1). This result demonstrates that, in response to denervation, skeletal muscle expresses the same *Scn5a* isoform expressed in the adult heart.

### 2.2. Skeletal Muscle Denervation Leads to Significant Changes in the Transcriptome

Classical studies on re-expression of *Scn5a* in denervated skeletal muscle were focused on the analysis of the channel properties and function. However, the mechanisms underlying this phenomenon remain largely unknown. To further understand at the molecular level how *Scn5a* re-expression is established, we first examined the changes in the transcriptome induced by denervation. Using gastrocnemius muscle from four rats, we compared mRNA levels from denervated and contralateral intact (control) muscle by RNA-sequencing (RNA-seq) analysis.

**Figure 2:**
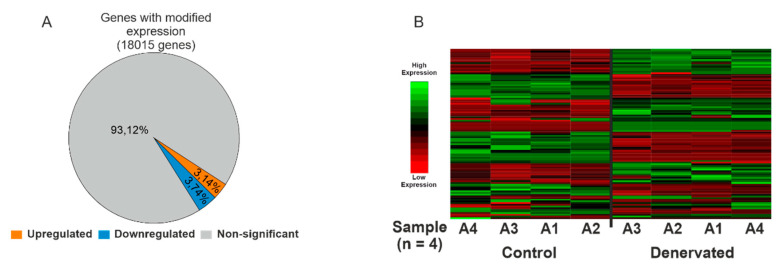
Differential RNA-seq analysis of denervated skeletal muscle compared to control, innervated muscle. (**A**) Pie Chart shows the percentage of genes with no significant expression changes (grey), significantly upregulated (orange) and significantly downregulated (blue). (**B**) Heatmap showing the 50 top upregulated and downregulated genes upon denervation. Each row represents the expression level of a particular gene in control (*n* = 4) and denervated samples (*n* = 4).

From a total of 32,254 annotated genes, we detected changes in the expression of 18,015 genes with DESeq2. Among these, only 6.88% were differentially expressed (DE, *p* < 0.001), being 3.14% upregulated and 3.74% downregulated (Figure 2A and Table S3). When we compared the expression levels of the fifty up and downregulated genes we observed that *Scn5a* was among the top fifty upregulated genes (Table S4). Importantly, we observed similar expression patterns within each group of samples (Figure 2B), indicating a good level of homogeneity among replicates regarding changes in the transcriptome induced by denervation.

### 2.3. Re-Expression of Scn5a Is Not Related to any Functional Annotation Based on Gene Ontology (GO) or Kyoto Encyclopedia of Genes and Genomes (KEGG) Pathways

**Figure 3:**
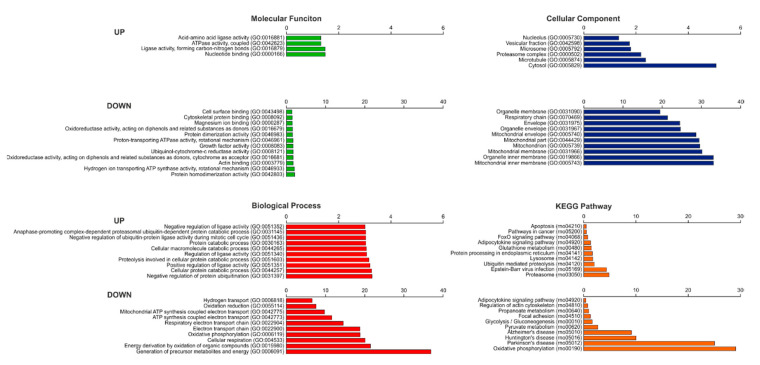
Gene Ontology (GO) enrichment annotation analysis of differentially expressed (DE) genes. GO categorization of DE genes in molecular functions (MF), cellular components (CC), biological processes (BP), and Kyoto Encyclopedia of Genes and Genomes (KEGG) Pathway (shown as -log_10_ *p*-value). Only the top ten significant upregulated and downregulated annotations are shown (*p* < 0.05).

To examine whether changes in *Scn5a* expression in denervated muscle could fall into a specific cellular function, we performed GO analysis of the DE genes. We categorized them by molecular functions (MF), cellular components (CC), biological processes (BP), and functional networks (KEGG; Kyoto Encyclopedia of Genes and Genomes) to determine which patterns were modified upon denervation (Tables S5–S12). To perform this analysis, we used DAVID web-based applications (https://david.ncifcrf.gov/ (accessed on 1 March 2021)). Significant annotations are shown in Figure 3.

We observed that, among the upregulated genes, the MF annotations showed an enrichment of nucleotide binding (GO:0000166), while protein homodimerization (GO:0042803) was enriched among the downregulated genes. Regarding the CC, upregulated genes are mostly cytosol components (GO:0005829), while downregulated genes are mainly mitochondrial inner membrane components (GO:0005743). These results suggest that denervation of skeletal muscle is associated with degradation processes and structural reorganization at the cellular level. In addition, alterations of the mitochondrial membrane components could be associated with mitochondrial dysfunction. In regard to the BP, we observed that, four days after denervation, catabolic processes are positively enriched, whereas the cell energy generation is negatively regulated. In agreement with this observation, the most downregulated BP are related to the generation of precursors of metabolites and energy (GO:0006091). Finally, analysis of the most significant changes on KEGG pathways showed that the most positively enriched pathway was the proteasome (rno03050), and the most negatively enriched pathway was oxidative phosphorylation (rno00190).

Despite being within the top upregulated genes, *Scn5a* was not included in any of the three categories of upregulated GO terms and neither in KEGG pathway annotations. These findings suggest that *Scn5a* gene re-expression in denervated muscle is not related to a general activation of a specific cellular pathway.

### 2.4. Scn5a TAD Neighboring Genes and Other Sodium Channels Are not Upregulated Upon Muscle Denervation

**Figure 4:**
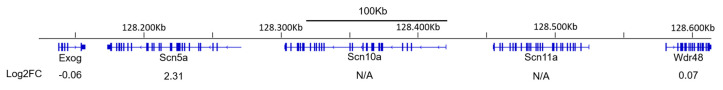
*Scn5a* is the only gene upregulated within the topological associated domain (TAD)**.** Limits of the TAD in which *Scn5a* is included, and changes in the expression of genes located within the TAD are shown as log2FC.

To investigate whether expression of the *Scn5a* gene upon denervation is caused by a regional transcriptional activation, we examined the changes in expression of *Scn5a* neighboring genes, focusing on those genes located within the topological associated domain (TAD) of *Scn5a*. Although Hi-C data from rat is not available, it is well known that TADs are highly conserved among species. Therefore, we set the limits of the *Scn5a* TAD based on mouse and human data [34,35] (Figure 4). From all genes identified within the *Scn5a* TAD (*Exog*, *Scn5a*, *Scn10a*, *Scn11a,* and *Wdr48*), only *Scn5a* showed a significant change in expression upon denervation (Figure 4). This result suggests that overexpression of *Scn5a* in denervated muscle is not related to a global transcription activation within this TAD region.

Next, we explored the possibility that re-expression of *Scn5a* could be associated with a general upregulation of genes encoding other sodium channel α or β subunits. However, we observed that only the transcript levels of *Scn5a* and *Scn1a* were significantly altered (Table 1).

To look into the possible factors intervening in the re-expression of *Scn5a*, we investigated which genes, among all the DE genes, correspond to transcription regulators. We imposed a threshold in the log2 fold change (FC) and selected those DE with an expression change ≥2 for the upregulated and ≤−2 for the downregulated genes (Table 1). Intriguingly, we observed that the mRNA levels of the TF *Gata4* are significantly upregulated upon skeletal muscle denervation. Since Gata4 has been shown regulate *Scn5a* transcription [32,33], this suggests that Gata4 could also be involved in *Scn5a* re-expression upon denervation. It is important to note that, consistently with Zaglia et al. (2009) [36] we could not detect Gata4 from total lysates in skeletal muscle samples by Western blot (Figure S1A). However, Gata4 was efficiently immunoprecipitated with α-GATA4 antibodies in both control and denervated muscle (Figure S1B), thereby confirming the presence of Gata4 protein in these samples.

We also examined potential epigenetic modifiers that could have a role in *Scn5a* re-expression. For this purpose, we imposed the same log2FC threshold as in the transcription regulator genes. However, in this case we only found significant changes for the class II histone deacetylase *Hdac4*, which was upregulated upon denervation (Table 1). Hdac4 is known to be upregulated during muscle denervation, and it is thought to be a key player in activity-dependent muscle remodeling [37,38].

### 2.5. Denervation Is Associated with an Enrichment of H3K27ac and H3K4me3 and Gata4 Binding at the Scn5a Promoter Region

**Figure 5:**
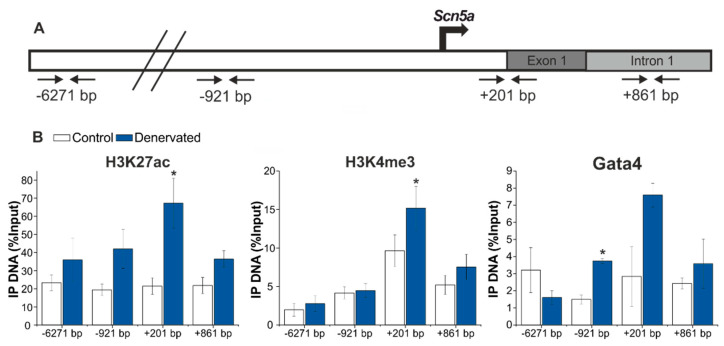
Enrichment of H3K27ac and H3K4me3 and binding of Gata4 near the *Scn5a* promoter in the denervated muscle. (**A**) Schematic view of the localization of the four primer pairs used by qPCR. Numbers indicate outermost left base pairs of amplicons. (**B**) Chromatin immunoprecipitation (ChIP) assays from control (white) and denervated (blue) samples using antibodies against H3K27ac, H3K4me3 and Gata4 and followed by qPCR. Results are shown as percentage of input (mean ± SE). H3K27ac (*n* = 6), H3K4me3 (*n* = 6) and Gata4 (*n* = 3). Significance was analyzed with *t*-test (* *p* < 0.05).

To further explore the molecular mechanisms associated with transcriptional activation of *Scn5a*, we studied epigenetic changes at the *Scn5a* promoter region. The *Scn5a* promoter region includes regulatory elements of the non-coding exon 1 as well as in the intron 1 [39]. We therefore examined the epigenetic profiles of Histone H3 lysine (K) 4 trimethylation (H3K4me3) and Histone H3 lysine (K) 27 acetylation (H3K27ac) across the promoter and first intronic regions of the *Scn5a* gene by chromatin immunoprecipitation (ChIP) analyses (Figure 5). H3K4me3 is a histone mark associated with transcriptionally active promoters, while H3K27ac is associated with active promoters and enhancers. Soluble chromatin from control and denervated muscle was prepared and incubated with H3K4me3 or H3K27ac antibodies (Figure 5). Analysis of the immunoprecipitated material by qPCR showed a significant enrichment of both H3K27ac (Figure 5, left) and H3K4me3 (Figure 5, middle) near the *Scn5a* promoter region (+201 bp) upon denervation. For H3K4me3, we also detected a small, but not significant enrichment in the intron 1 region (+861 bp), which would be in agreement with the localized action of this epigenetic mark surrounding active promoters, and not in regions further upstream. Overall, these results suggest that *Scn5a* re-expression in denervated muscle is associated with increased H3K4me3 and H3K27ac marks at the *Scn5a* promoter region, near the transcription start site (TSS).

To study whether Gata4, which was found to be upregulated in our RNA-seq analysis, would be also involved in the transcriptional activation of the *Scn5a* gene in denervated muscle, we performed ChIP-qPCR using specific antibodies against Gata4. Similar to the previously described binding of Gata4 at the *Scn5a* promoter in human heart, we observed robust binding of Gata4 in the promoter regions in denervated skeletal muscle (Figure 5, right). These results suggest that the TF Gata4 contributes to *Scn5a* re-expression in skeletal muscle in response to denervation.

### 2.6. Scn5a Re-Expression in Skeletal Muscle Is Associated with Activation of a Super Enhancer Downstream of the Scn5a Gene

To further examine the changes on histone modifications associated with muscle denervation, we studied the H3K27ac profiles genome-wide by ChIP-seq experiments. Analysis of the ChIP-seq data showed a total of 55,089 peaks in both control and denervated samples and a similar distribution of these peaks within the genomic annotations in both conditions (Figure S2). We also observed a similar number of peaks in control and denervated samples, although 19.47% and 24.28% of the total peaks were condition-specific for control and denervated muscle, respectively (Figure S3).

**Figure 6:**
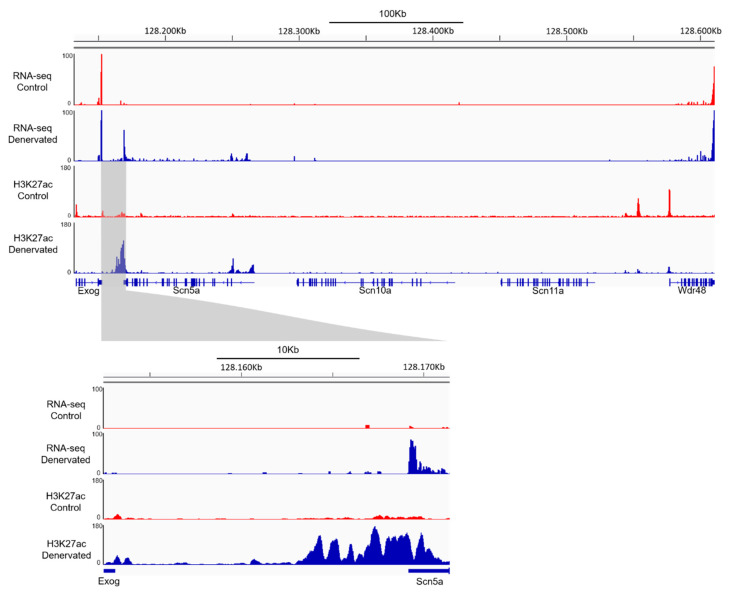
Denervation induces the activation of a super enhancer downstream of *Scn5a*. Integrative Genome Viewer (IGV) view of the H3K27ac distribution pattern in the *Scn5a* TAD and the reads of RNA-seq data in control and denervated gastrocnemius muscle samples. Expanded view of the super enhancer region located between *Exog* and *Scn5a* gene (bottom).

To perform a differential enrichment analysis of control and denervated samples, we imposed a log2 FC threshold of 2 and −2 for the regions showing enrichment or reduction, respectively, of the H3K27ac marks. We observed significant changes in H3K27ac patterns in a total of 1403 genomic regions (Table S13), 67.07% showing an increase and 32.93% a decrease in the H3K27 acetylation levels. From all the differential enriched regions, only 170 were localized in nearby genes. We analyzed our RNA-seq data and found that, from these 170 genes showing significant changes in H3K27ac patterns, 63.53% were upregulated and 36.47% were downregulated in denervated, respect to control muscle (Table S14). Focusing on the *Scn5a* TAD, we observed that only 6 regions were identified in the differential enrichment analysis. All these regions were localized downstream of the *Scn5a* gene (Figure 6). As mentioned above, *Scn5a* is the only gene in this TAD showing significant increase in gene expression, suggesting that the H3K27ac enrichment in this region is only affecting *Scn5a* expression.

Then, we performed a super enhancer analysis using a proprietary algorithm (Active Motif), categorizing a total of 1019 super enhancers, from which 502 were induced and 517 were repressed upon denervation (Table S15). Among those, the enriched H3K27ac region downstream of *Scn5a* (Figure 6) was identified as the super enhancer with the highest enrichment (Table 2 and Figure 6). Importantly, this region has been recently described as a super enhancer that regulates *Scn5a* gene expression in mouse heart [40]. These results suggest that, upon denervation of skeletal muscle, re-expression of *Scn5a* involves similar molecular mechanisms than those responsible for the expression of *Scn5a* in the adult heart.

## 3. Discussion

This study reveals that the molecular mechanisms associated with re-expression of the *Scn5a* gene in denervated skeletal muscle resemble those that activate *Scn5a* transcription in cardiac tissue. Using rat gastrocnemius muscle as a model, we show that denervation upregulates the expression of the adult *Scn5a* isoform that is mostly expressed in adult cardiac myocytes. RNA-sequencing analysis of control and denervated samples demonstrates that denervation induces significant changes on the transcript levels of 6.88% of the *Rattus norvegicus* genes. *Scn5a* emerges as one of the most significant upregulated genes, although GO analysis of DE genes suggests that *Scn5a* gene re-expression upon denervation is not related to a general activation of a specific cellular pathway. In agreement with this analysis, we did not find significant expression changes of other sodium channel α or β subunits apart from *Scn1a*, which is significantly downregulated upon denervation. ChIP-qPCR experiments suggest that *Scn5a* re-expression is associated with an enrichment of H3K4me3 and H3K27ac histone marks and binding of the TF Gata4 at the promoter region. Importantly, ChIP-seq analysis of H3K27ac shows that denervation is associated with activation of a super enhancer recently described to regulate *Scn5a* expression in cardiac tissue.

*Scn5a* is normally expressed in neonatal skeletal muscle, but just after birth this expression rapidly decreases, and the adult muscle expresses the *Scn4a* gene instead [12]. Although it has long been known that denervation of skeletal muscle induces re-expression of *Scn5a*, the specific isoform expressed under these conditions was never identified. Here, we show that, unlike other tissues in which *Scn5a* is abnormally expressed (such as breast cancer cells) [41], rat denervated gastrocnemius muscle expresses the adult cardiac *Scn5a* isoform.

Our RNA-seq analysis of control and denervated samples only shows changes in the transcript levels of two sodium channel α or β subunits: *Scn5a*, which is upregulated, and *Scn1a*, which is downregulated. A decrease in *Scn1a* mRNA could be a consequence of the degeneration of the nerve terminals after denervation. Although mRNA axonal transport and local protein synthesis are well-known mechanisms in neurons [42], to date, there is no direct evidence for the presence of *Scn1a* mRNA in the motor neuron terminal. Nevertheless, it has been shown that *Scn10a* mRNA, encoding Na_V_1.8, can be peripherally transported from the neuronal cell bodies to the sciatic nerve [43]. Similarly, we may then speculate that *Scn1a* mRNA transcripts, present in the nerve terminals, would disappear upon denervation and this could account for the diminished gene expression. Of note, our observation that *Scn4a* transcript levels are not significantly affected by denervation is in agreement with early studies showing that *Scn4a* (*SkM1*) expression persists upon denervation [20].

Our comparison of the RNA-seq and ChIP-seq data within the *Scn5a* locus shows that the synthesis of *Scn5a* mRNA transcripts in response to denervation correlates with the enrichment of H3K27ac peaks at a downstream *Scn5a* region. This region was recently described by Man and colleagues as an enhancer cluster with features of a super enhancer that regulates the topology of the *Scn5a*-*Scn10a* locus [40]. They reported that deletion of the enhancer cluster only leads to a reduction of *Scn5a* expression, and to a lesser extent *Scn10a*, from all the genes included in the TAD. In agreement with their findings, our RNA-seq data show that activation of the super enhancer upon denervation only affects the expression of the *Scn5a* gene within the TAD. This result suggests that RE-promoter interactions within the *Scn5a*-*Scn10a* locus are conserved between cardiac tissue and denervated muscle, even across species, and further demonstrates that overexpression of *Scn5a* is not related to a global transcription activation within this TAD region. In the absence of rat heart or denervated human skeletal muscle data, we performed a side by side comparison of the H3K27ac pattern from this region between human left ventricle and denervated rat skeletal muscle. These alignments reveal an extensive degree of overlapping histone marks (Figure S4). However, some tissue- and/or species-specific differences can be pointed out. Most notably, the RE-9 as defined by Man et al. [40] is completely absent in denervated muscle. Other peaks, however, do overlap to a large extent within the broader region encompassing RE 6-9. According to Man et al. [40] heterozygous removal of this entire region completely abolishes the *Scn5a* expression of the modified allele in the mouse heart, and it produces conduction slowing. In this sense, it would be interesting to analyze denervated muscle from these animals and examine whether this region also controls *Scn5a* expression in this tissue.

In agreement with previous reports showing a regulatory role of Gata4 in *SCN5A* expression in the human heart, our data provides important evidence suggesting that Gata4 contributes to the transcriptional activation of the *Scn5a* gene in denervated muscle. RNA-seq analysis shows a significant increase of Gata4 expression in denervated muscle. Furthermore, the ChIP-qPCR experiments show an enrichment in Gata4 binding at the *Scn5a* promoter region in response to denervation, indicating the presence of this protein in our experimental conditions. This was further confirmed by immunoprecipitation followed by Western blot analysis (Figure S1). The presence of Gata4 was observed in both control and denervated muscle extracts. Previously studies by Zaglia et al. (2009), showed that Gata4 was not detectable by immunostaining in normal and regenerating skeletal muscle cryosections [36]. Also, van Tuyn et al. (2006) showed the absence of *Gata4* mRNA in normal skeletal muscle [44]. We did not detect Gata4 protein in total protein extracts from either control or denervated skeletal muscle (Figure S1). It is possible thus, that levels of Gata4 are too low to be detected by immunostaining or in total protein extracts, but that the concentration reached by the IP made it apparent in our Western blot analysis. Also, our RNA-seq experiments showed the differential expression of GATA4 in denervated muscle respect to control, but not basal RNA levels. Thus, it is likely that RNA levels of GATA4 in the control gastrocnemius is low and hardly detected as observed by van Tuyn et al. [44]. Alternatively, the discrepancy that seems to appear between theirs and our results could be due to the differences between gastrocnemius and vastus lateralis muscles. On the other hand, although immunoprecipitation allowed us to confirm the presence of Gata4 at the protein level, this low level of detection is not suited for accurate quantitative estimates of protein abundance. Thus, whether this augmented Gata4 binding is the consequence of the increased *Gata4* mRNA levels observed in our RNA-seq experiments, remains to be determined. Alternatively, this enhanced binding can also be explained by an increased recruitment of existing Gata4 protein to the promoter region. Gata4 is a member of the Gata family of zinc-finger TF that is widely expressed in the developing and adult heart. It is considered a master regulator of transcriptional networks and plays a critical role in cardiac differentiation and morphogenesis [45]. Accordingly, Gata4 null mice show cardiac defects that result in embryonic lethality [46] and genetic variants affecting GATA4 activity have been associated with a range of cardiac defects including right ventricular hypoplasia and cardiomyopathy [47]. Additionally, it has been reported that *Gata4* +/− mice show short PR intervals, further underscoring the critical role of Gata4 in the development of the atrioventricular cardiac conduction system [48]. Mechanistically, Gata4 has been shown to synergistically interact with other TF, including Nkx2-5, TBX5, and MEF2, among others [30,49]. ChIP-seq studies have identified co-occupancy of cardiac enhancers by these TF together with the histone acetyltransferase KAT3B and have demonstrated that Gata4 activates gene expression by promoting H3K27ac deposition [49,50].

To our knowledge, our results point out, for the first time, to a central role of Gata4 in the regulatory transcriptional events associated with muscle denervation. Based on previous findings, we could also speculate that Gata4 may be interacting with other TF to activate *Scn5a* gene expression. Although we found that Gata4 is the most upregulated cardiac TF, Tbx5 expression is also slightly increased upon denervation and could be interacting with Gata4 at the promoter region. In this regard, our preliminary data indicate that Gata4 and Tbx5 synergize in the activation of the human *SCN5A* promoter (not shown). Our data also raises the possibility that Gata4 plays a major role in the super enhancer activation at the *Scn5a* locus. In this sense, further ChIP-seq studies using antibodies for Gata4 or other factors are required to ultimately uncover the molecular mechanism triggering hyperacetylation of this super enhancer.

Altogether, our data demonstrates that denervation of skeletal muscle induces upregulation of *SCN5A* gene expression via transcriptional regulatory mechanisms that resemble those controlling its expression in cardiac tissue. This suggests that denervated muscle is a suitable model to study the regulation of *SCN5A* gene expression, which is fundamental to unveil novel etiologies of sodium channel-related arrhythmias.

## 4. Materials and Methods

### 4.1. Rat Skeletal Muscle Denervation and Sample Collection

Experiments were performed on four adult young male Sprague–Dawley rats (60 to 70 days post-natal, Charles River, L’Arbresle, France). Rats were cared for in accordance with the guidelines of the European Community’s Council Directive of 24 November 1986 (86/609/EEC) and the Spanish Royal Decree 53/2013 for the humane treatment of laboratory animals. Animals were anesthetized with 2% tribromoethanol (0.15 mL/10 g body weight intraperitoneal). Then, the lumbosacral area was shaved, and the skin and muscles were gently dissected until the visualization of the sciatic nerve. We sectioned the sciatic nerve 5 mm apart from the column and then the area was sutured to close the incision. Four days after denervation, rats were euthanized by exsanguination while deeply anesthetized. Denervated and contralateral innervated gastrocnemius muscles were excised and dissected on a Sylgard-coated Petri dish containing normal Ringer solution and continuously bubbled with 95% O_2_-5% CO_2_. Samples were then frozen in liquid nitrogen and stored at −80 °C until use. These experimental procedures were reviewed and approved by the Ethics Committee of the Rovira i Virgili University, Reus, Spain (Reference number: 0233).

### 4.2. RNA Isolation, cDNA Synthesis, and Scn5a Sequencing

We disrupted 30 mg of denervated gastrocnemius muscle samples with a glass dounce homogenizer in RLT buffer supplemented with 0.01% of β-mercaptoethanol, and purified total RNA using the RNA Fibrous Tissue Mini Kit (Qiagen, Hilden, Germany) according to the manufacturer’s specifications. To avoid contamination with genomic DNA, we included a step of DNase I treatment. After reverse transcription of 1 µg of total RNA using QuantiTect Reverse Transcription Kit (Qiagen) we PCR-amplified the *Scn5a* cDNA with specific primer pairs (Table S1) using the GoTaq G2 Colorless Master Mix (Pomega, Madison, WI, USA) and Veritiy 96 Well Thermal Cycler (Applied Biosystems, Foster City, CA, USA). PCR products were purified with ExoSAP-IT (ISOGEN Life Science, Utrecht, Netherlands) and directly sequenced in both directions with BigDye Terminator v3.1 cycle sequencing and 3130xl Genetic analyzer (Applied Biosystems). Sequences quality was analyzed with Sequencing Analysis 5.3.1 (Applied Biosystems) and then compared to the *Scn5a* adult exon 6 sequence (ENSRNOT00000064555.3) using SeqScape v2.6 (Applied Biosystems).

### 4.3. RNA-Seq and Data Analysis

Total RNA from control and denervated muscle samples from four animals was extracted as described in Section 4.2. RNA quality was analyzed with Agilent 2100 Bioanalyzer (Agilent Technologies, Santa Clara, CA, USA) using the RNA Nano Kit 6000 (Agilent technologies). For library preparation, we only selected control and denervated samples from the same animal with an RNA Integrity Number (RIN) ~10. Library construction and data analysis were performed by the Center for Genomic Regulation (CRG, Barcelona, Spain). RNA-seq libraries were constructed with TruSeqv4 mRNA Sample Preparation Kit (Illumina, San Diego, CA, USA) and sequenced (50-base paired-end) in a HiSeq 2500 platform (Illumina). On average, we obtained 35 million paired reads per sample. Reads quality was evaluated by checking the presence of ribosomal contamination and their Sanger qualities, and then mapped to rat genome (Rnor_6.0) using TopHat v2.0.14. Univocally mapped tags were counted and assigned to annotated genes using HTSeq-count v.0.6.1p1. DE genes were detected using DESeq2. DE genes were considered if Benjamini–Hoechberg (BH) adjusted *p*-value <0.001 and divided in up and down-regulated according to the fold change and used for gene set enrichment analysis with DAVID web application (https://david.ncifcrf.gov/summary.jsp (accessed on 1 March 2021)) was done.

### 4.4. Immunoprecipitation Experiments and Western Blot

We disrupted 40 mg of heart, control and denervated gastrocnemius muscle samples using the TissueLyser LT (Qiagen) and stainless-steel beads in tissue lysis buffer (10% of glycerol, 1% of SDS and 50 mM of Tris-HCl pH 6.8, protease inhibitor cocktail (Roche, Madrid, Spain)). Samples were then sonicated to reduce the viscosity and centrifuged 15 min at 16,000× *g*. Protein quantification was performed with the Pierce BCA Protein Assay (Thermo Scientific, Waltham, Massachussets, US). One milligram of total lysate was immunoprecipitated with α-GATA4 polyclonal antibodies (PA5-29663, Thermo Scientific) in immunoprecipitation buffer (50 mmol/L Tris HCl pH 7.4, 150 mmol/L NaCl, 1 mmol/L EDTA, 1% NP-40 and Protease Inhibitors Cocktail (Roche)) overnight at 4 °C. Protein-A beads were extensively washed, and immunocomplexes were analyzed by Western blot with monoclonal α-Gata4 antibodies (SC-25310, Santa Cruz Biotechnology, Dallas, TX, US) and the α-mouse secondary antibody conjugated with horse radish peroxidase (115-035-003, Jackson Immuno Research, Cambridgeshire, UK). 1% of total protein was used as input.

### 4.5. ChIP-qPCR Assays

ChIP assays were performed using control and denervated gastrocnemius samples from the same animal following the protocol described by Tarradas et al. [32]. Antibodies used for immunoprecipitation were α-H3K4me3 (07-473, Millipore, Burlington, MA, USA), α-H3K27ac (ab4729, Abcam, Cambridge, UK) and α-Gata4 (ma5-15532, Thermo Fisher). We analyzed the immunoprecipitated material by qPCR using primers specific for four regions of the rat *Scn5a* locus (Table S2). Results are shown as percentage of input.

### 4.6. ChIP-Seq Assays

Denervated and contralateral innervated muscle samples from one animal were sent to Active Motif’s epigenetic services for ChIP-seq experiments for H3K27ac and data analysis. Briefly, ChIP-seq libraries were sequenced on NextSeq 500 (Illumina) and sequencing reads were mapped to the reference genome using the BWA algorithm with default settings. Duplicate reads were removed and only reads that passed the Illumina’s purity filter, aligned with less than 2 mismatches, and mapped uniquely to the genome were used in subsequent analysis. ChIP peaks were called by a combination of two peak callers MACS/MACS2 and SICER. Finally, peaks were annotated and information regarding genes and other genomic features was extracted. For the super enhancers’ analysis, Active Motif uses their own software. MACS or SICER peaks generated by the ChIP-seq analysis are merged if their inner distance is equal or less than 12.500 bp. Then, the merged peaks with strongest signals (top 5%) are identified as super enhancers.

### 4.7. Statistical Analysis

Results are represented as mean ± SEM. Statistical analysis was performed using OriginPro 8 (OriginLab, Wellesley Hills, MA, USA). For ChIP-qPCR experiments, we compared each region between control and denervated samples with a T-test. Differences were considered significant at *p* ≤ 0.05 (*).

## Figures and Tables

**Table 1:** Gene expression changes in denervated compared to control, innervated, muscle in the indicated categories. For each gene, the log2FC, and significance (*p*-value) are indicated.

	Gene	Log2 FC	*p*-Value
α sodium channel subunits	*Scn1a*	−1.98	4.57 × 10^−4^
	*Scn2a*	−0.25	NS
	*Scn3a*	0.17	NS
	*Scn4a*	0.13	NS
	*Scn5a*	2.31	4.29 × 10^−5^
	*Scn7a*	−1.08	NS
	*Scn8a*	0.05	NS
	*Scn9a*	−0.25	NS
β sodium channel subunits	*Scn1b*	0.34	NS
	*Scn2b*	0.11	NS
	*Scn3b*	−0.93	NS
	*Scn4b*	−0.45	NS
Transcription regulators	*Arx*	−2.37	2.58 × 10^−16^
	*Fos*	−2.02	2.58 × 10^−15^
	*Foxp3*	−2.07	1.53 × 10^−4^
	*Gata4*	2.36	2.27 × 10^−5^
	*Msx3*	−2.43	1.25 × 10^−5^
	*Myog*	2.93	3.85 × 10^−61^
	*Nfkb2*	2.04	1.04 × 10^−22^
	*Nlrc5*	2.55	2.06 × 10^−13^
	*Nr4a1*	−3.16	2.15 × 10^−33^
	*Nr4a2*	−2.4	1.49 × 10^−9^
	*Perm1*	−3.69	2.49 × 10^−33^
	*Runx1*	2.76	1.16 × 10^−16^
	*Tgif1*	2.06	3.16 × 10^−20^
	*Tmem229a*	−2	5.16 × 10^−4^
Epigenetic modifiers	*Hdac4*	2.23	1.08 × 10^−12^

**Table 2:** Top ten activated super enhancers upon denervation. Super enhancers were sorted by the log2FC of the average value of H3K27ac enrichment. Change in H3K27ac enrichment in the super enhancer delimited region (log2FC ChIP-seq), effect on the expression of the genes localized near the super enhancer region (log2FC RNA-seq) and their significance (*p*-adj RNA-seq).

Chromosome	Start	End	Gene	Log2fc ChIP-seq	Log2fc RNA-seq	*p*-Adj RNA-seq
8	128,153,123	128,170,975	*Exog*	3.27	-	-
			*Scn5a*	3.27	2.31	4.29 × 10^−5^
4	97,879,010	97,895,598		2.33	-	-
2	188,844,059	188,877,449	*Kcnn3*	1.89	2.15	3.32 × 10^−20^
2	34,586,496	34,618,138	*Adamts6*	1.85	0.16	NS
9	119,081,166	119,099,933	*Dlgap1*	1.84	0.75	NS
			*LOC102555426*	1.84	-	-
2	188,892,238	188,924,410	*Kcnn3*	1.83	2.15	3.32 × 10^−20^
3	151,407,846	151,445,826	*Uqcc1*	1.76	0.06	NS
1	140,978,031	141,044,113	*Abhd2*	1.67	2.98	7.94 × 10^−23^
1	175,572,393	175,629,876	*LOC103691204*	1.61	-	-
			*Ampd3*	1.61	1.94	3.46 × 10^−4^
			*rnf141*	1.61	0.33	NS
2	203,895,667	203,947,339		1.60	-	-

## Data Availability

ChIP-seq and RNA-seq data presented in this study are openly available European Nucleotide Archive under the accession number PRJEB41483.

## Supplementary Materials

The following are available online at https://www.mdpi.com/1422-0067/22/5/2755/s1, including Supplemental Tables and Figures.

## Abbreviations

BP	Biological process
BrS	Brugada syndrome
CC	Cellular component
ChIP	Chromatin immunoprecipitation
DE	Differentially expressed
FC	Fold change
H3K27ac	Histone H3 lysine (K) 27 acetylation
H3K4me3	Histone H3 lysine (K) 4 trimethylation
KEGG	Kyoto Encyclopedia of Genes and Genomes
MF	Molecular function
RE	Regulatory elements
RNA-seq	RNA sequencing
TAD	Topological associated domain
TF	Transcription factor
TSS	Transcription start site
TTX	Tetrodotoxin
VGSC	Voltage-gated sodium channel/s
