# Supplementary material for: Epigenetic Changes Governing Scn5a Expression in Denervated Skeletal Muscle
Source: Int J Mol Sci. 2021 Mar 9;22(5):2755. doi: 10.3390/ijms22052755 (PMC7963191; doi:10.3390/ijms22052755)
Supplement: Supplementary file 1 [file ijms-22-02755-s001.zip › Supplementary material/Supplementary material Carreras et al.docx]

**Supplementary material**

**Supplementary tables**

**Table S1.** Primers used for sequencing *Scn5a* cDNA from skeletal denervated muscle

| Primer | Sequence (5' to 3') | Regions covered | Amplicon (bp) |
| --- | --- | --- | --- |
| 1 | Fw: CTGCCCCAAGCCCTACG | Exon 1 to 5 | 730 |
|  | Rv: GAAGTCTAGCCAGTTCCACGG |  |  |
| 2 | Fw: CCTTCACTGCCATCTACACCT | Exon 5 to 7 | 368 |
|  | Rv: GGTGAAGTTACGCACACACT |  |  |
| 3 | Fw: AGTGTCTTTGCCCTCATTGG | Exon 7 to 9 | 358 |
|  | Rv: TCTGCTGGTATAGGCGTTCC |  |  |
| 4 | Fw: GCAGGTGAGAACCCAGACC | Exon 9 to 10 | 310 |
|  | Rv: CCTCGTGTTCCTTCTTGAGC |  |  |
| 5 | Fw: TACGAGGAGCAAAACCAAGC | Exon 10 to 12 | 404 |
|  | Rv: GTGCTGTTCTCGTCATCTGC |  |  |
| 6 | Fw: AGGGAGCATTTTCACGTTCC | Exon 12 to 13 | 366 |
|  | Rv: GGAGCCTGAGGTGTCAGC |  |  |
| 7 | Fw: ATGGGGTGGTTTCCTTGC | Exon 12 to 14 | 268 |
|  | Rv: CAGCATGGTGGACACTTCC |  |  |
| 8 | Fw: CCATCCCAGCGCCCAAG | Exon 12 to 16 | 916 |
|  | Rv: GCTGATGCGGTGCCTCA |  |  |
| 9 | Fw: CATCCTCAGTCTCATGGAGCTG | Exon 15 to 17 | 850 |
|  | Rv: CTCTGTGCCAAGGCTGTTCTC |  |  |
| 10 | Fw: CCCACAGCCAGCTGCCCAG | Exon 17 to 22 | 771 |
|  | Rv: GTGCACGCAGTGTCCTCAGTG |  |  |
| 11 | Fw: CTGCTGGAGTACGCGGAC | Exon 21 to 27 | 900 |
|  | Rv: GAGGAACATGATGGTGACATCG |  |  |
| 12 | Fw: CCAGCAGAAGAAAAAGTTAGGG | Exon 25 to 28 | 901 |
|  | Rv: GTTCTCGAGGATGATGGCGATG |  |  |
| 13 | Fw: GTTCCAGATCACCACATCAGC | Exon 28 | 834 |
|  | Rv: GTGACGCTGTCGTAGGAC |  |  |
| 14 | Fw: GCCTTCCGGAGGCACC | Exon 28 to 3' UTR | 907 |
|  | Rv: CCCTAGCATGTGAAGTGTGCTC |  |  |
| 15 | Fw: CATCAGCAGTTCACACTGCCTC | 3' UTR | 912 |
|  | Rv: CCCTGAAAAAGCCTTCTCAGAAG |  |  |
| 16 | Fw: GGGACTCCAACTCCTCTTCAG | 3' UTR | 902 |
|  | Rv: CTAGCTTGCTCCTCCCCTCAG |  |  |

**Table S2.** Primers used for ChIP-qPCR analysis.

| Position relative to TSS | Start | End | Sequence (5' to 3') |
| --- | --- | --- | --- |
| -6271 bp | chr8:128,272,910 | chr8:128,27,2761 | Fw: TCAAAATCAGCGGCATCCAG |
|  |  |  | Rv: GTGGGAATTGGACCTGGGTA |
| -921 bp | chr8:128,267,560 | chr8:128,267,391 | Fw: TGGGACTTGGTATCCCTCTG |
|  |  |  | Rv: GCCATGCTGACACATGAACT |
| +201 bp | chr8:128,266,438 | chr8:128,266,309 | Fw: CCCAACTTTCCTCCCCGAT |
|  |  |  | Rv: TCAGAGACGAACAGGGGAAC |
| +861 bp | chr8:128,265,778 | chr8:128,265,621 | Fw: TGCTTTGACCTGGACAGACT |
|  |  |  | Rv: TTACCAGTCCCCACCACATC |

**Table S3.** Differential expression denervated vs control samples (excel file).

**Table S4.** Top 50 upregulated/downregulated genes in denervated vs control samples (excel file).

**Table S5.** GO analysis_Biological Process UP (text file).

**Table S6**. GO analysis_Biological Process DOWN (text file).

**Table S7.** GO analysis_ Cellular Component UP (text file).

**Table S8.** GO analysis_ Cellular Component Down (text file).

**Table S9.** GO analysis_ Molecular Function UP (text file).

**Table S10.** GO analysis_ Molecular Function DOWN (text file)

**Table S11.** Analysis KEGG pathways UP (text file).

**Table S12.** Analysis KEGG pathways DOWN (text file).

**Table S13.** ChIP-seq H3K27ac Top differential sites (excel file).

**Table S14.** Comparison of ChIP-seq H3K27ac peaks with RNA-seq (excel file).

**Table S15.** ChIP-seqH3K27ac SE_Mergedregs. Superenhancer analysis (excel file).

**Supplementary figures**


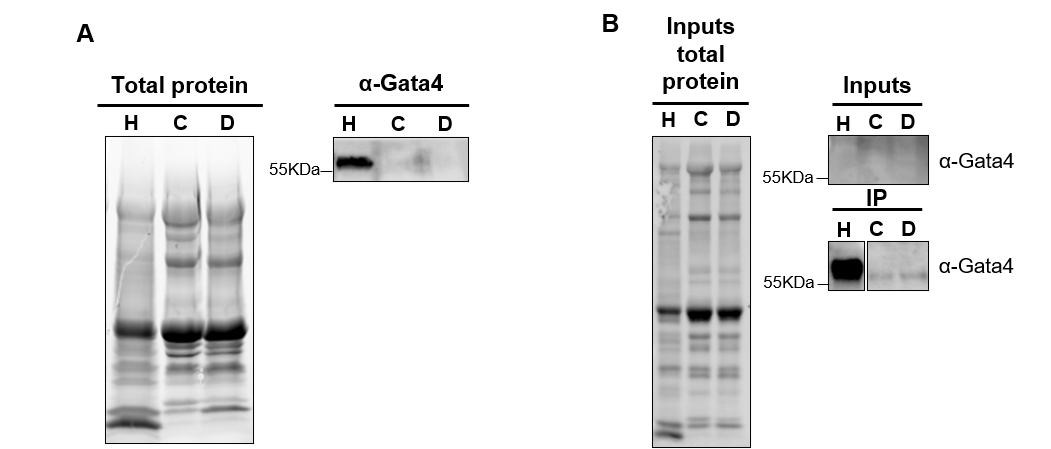


**Figure S1. Gata4 is immunoprecipitated from protein lysates of control and denervated skeletal muscle samples. A.** Total protein stain-free gel (left) and western blot (right) performed with 50 µg of total protein from heart (H), control (C) and denervated (D) muscles and analyzed with α-Gata4 antibodies.  **B.** Total protein (left) and western blot (right) of total lysates from heart, control and denervated samples immunoprecipitated with α-Gata4 antibodies.**.**


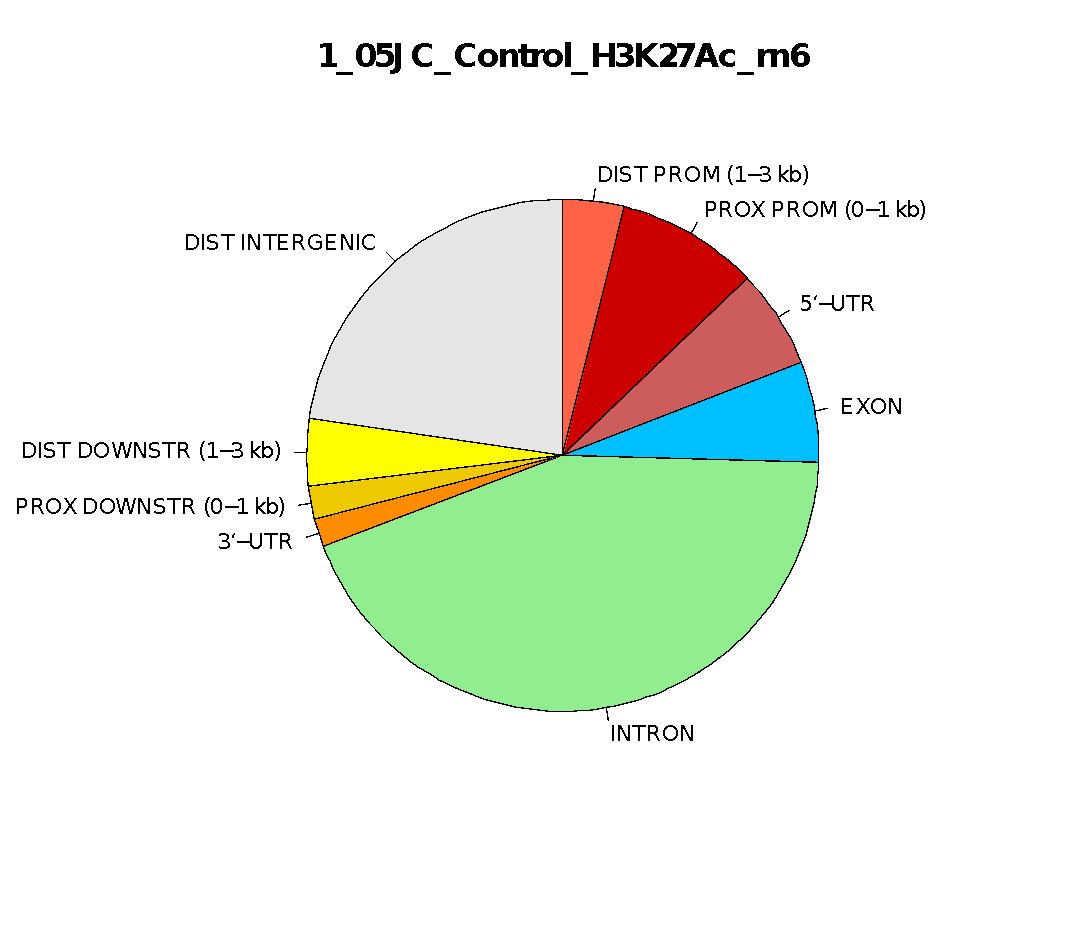


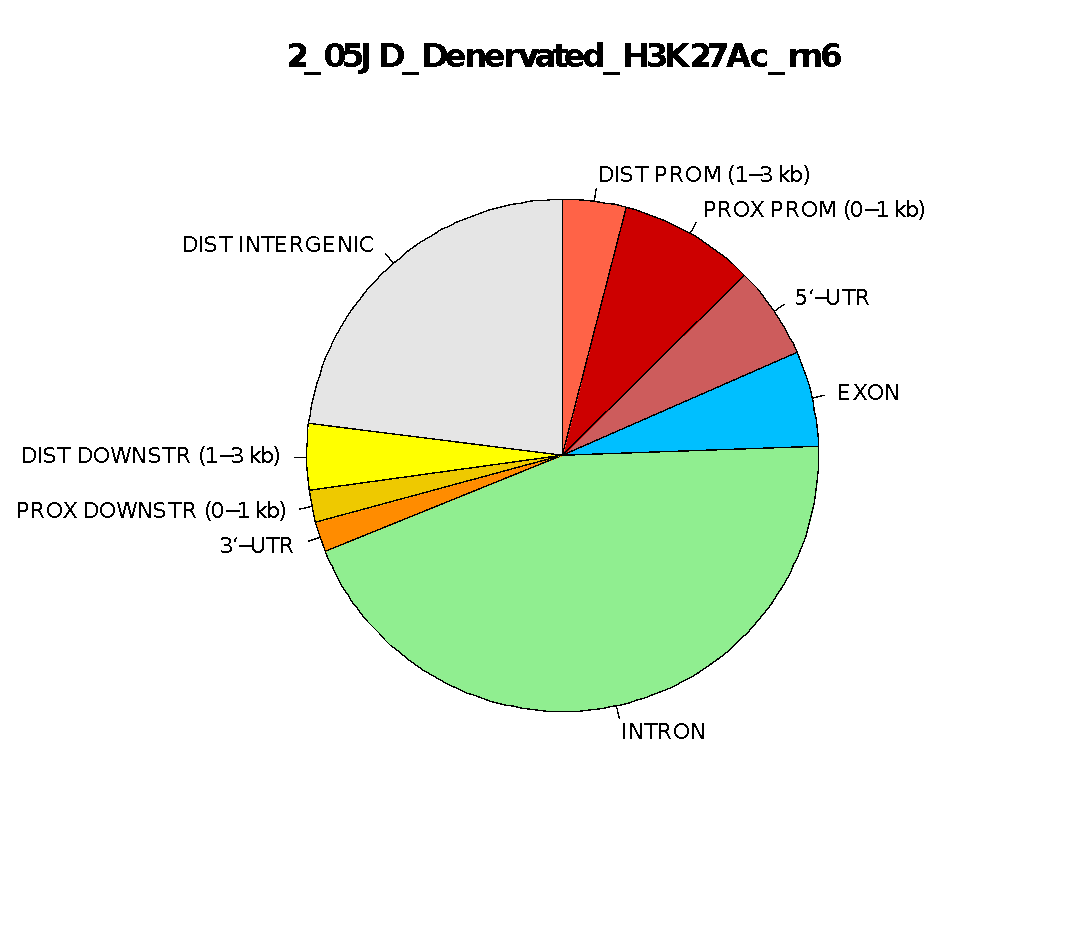


**Figure S2.** **Pie charts showing distribution of ChIP-seq H3K27ac peaks relative to genomic annotations in control and denervated samples.**

**
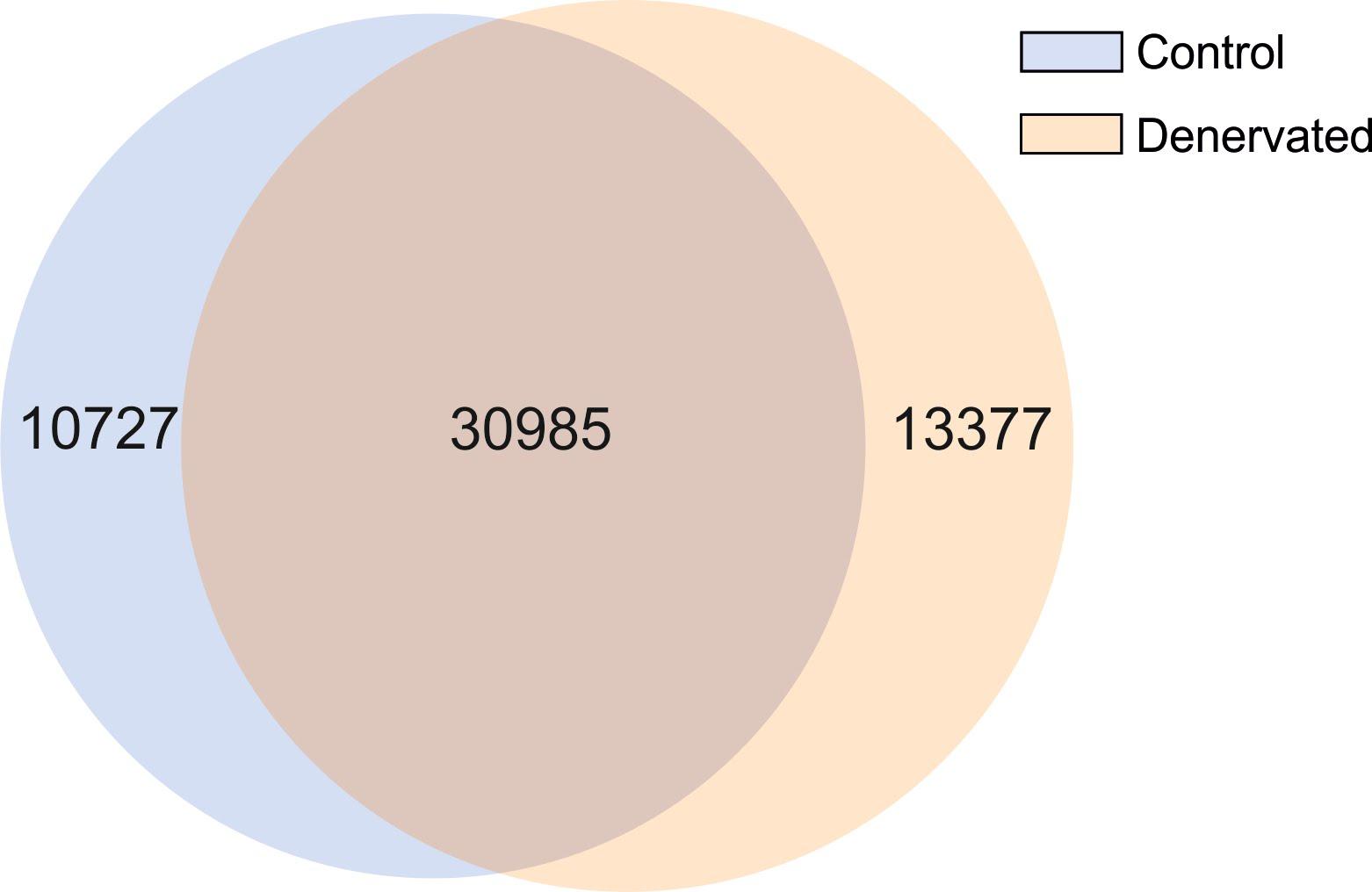
**

**Figure S3.** **ChIP-seq H3K27ac merged regions.** Venn diagram showing the overlapping peaks between control and denervated samples.

**
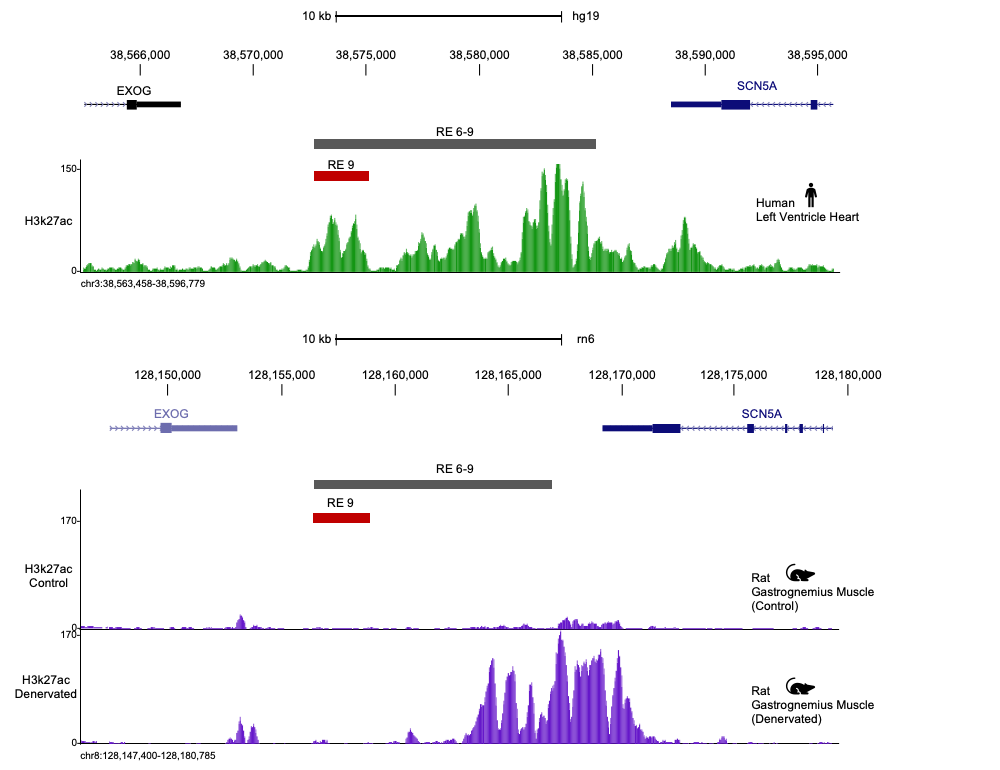
**

**Figure S4.** **Comparison of the H3K27ac peaks at the super enhancer between human left ventricle and rat denervated muscle.** UCSC browser view of the intergenic region between *SCN5A* and *EXOG* in human (hg19, top) and rat (rn6, bottom) genomes. The super enhancer is only active in cardiac and cardiac-like conditions, as delineated by H3K27ac ChIP-seq from human left ventricle (Ensembl Regulatory Build) and denervated rat gastrocnemius muscle. Regulatory elements (RE6-9) described in [36] are shown at the top of ChIP-seq data as tracks.
